# Rising Breast Cancer Incidence and Poor Outcomes in Young Women: A Retrospective Study

**DOI:** 10.1155/tbj/5584726

**Published:** 2026-02-24

**Authors:** Jennifer Den, Nicole Nelson, Raj Vaghjiani, Douglas Tyler, V. Suzanne Klimberg

**Affiliations:** ^1^ Department of General Surgery, The University of Texas Medical Branch, Galveston, Texas, USA, utmb.edu

**Keywords:** breast cancer, incidence, young women

## Abstract

**Background and Aims:**

Recent studies suggest a rise in breast cancer (BC) among young women. We analyzed BC incidence patterns among women aged 18–40 in the TriNetX network from 2014 to 2023, stratifying by age, race, and ethnicity. We also examined mortality and local recurrence (LR) in women aged 18–40 diagnosed with primary BC.

**Methods:**

This retrospective study used data from the TriNetX network. Female patients aged 18–40 were identified, and the incidence proportion of primary BC from 2014 to 2023 was assessed using the TriNetX Incidence and Prevalence Analytics function, with stratification by age, ethnicity, and race. A second cohort of women aged 18–40 with a primary BC diagnosis was created to evaluate mortality and LR over the same period. All analyses were descriptive.

**Results:**

Among the 18,250,987 women aged 18–40 years in the TriNetX network, the incidence proportion of BC increased from 635 cases in 2014 to 4475 in 2023. White women accounted for the greatest number of BC cases; however, increasing incidence proportions were also observed among American Indian/Alaskan Native (AI/AN), Asian, and Native Hawaiian/Pacific Islanders (NHPIs) women over the last four years. Non‐Hispanic women initially had higher incidence proportions but were surpassed by Hispanic women in 2022. Among 38,683 women aged 18–40 with primary BC, both mortality (0.09%–0.851%) and LR (0.3%–2.9%) increased over the study period.

**Conclusion:**

Within the TriNetX network, BC incidence proportion among women aged 18–40 demonstrated an upward trend from 2014 to 2023, particularly among non‐Hispanic White women 30–39 years old. An uptrend was also seen in young AI/AN, Asian, NHPI, and Hispanic women. In addition, increased documented mortality and LR were also observed. These findings underscore the importance of further research to understand these trends and develop diagnostic and therapeutic approaches tailored to younger patients.

**Highlights**
• The incidence proportion of BC among young women 18–40 years old in the TriNetX network has increased over the past decade.
• Young non‐Hispanic White women between 30 and 39 years old had the greatest incidence proportion of BC.
• Over the past 4 years, an uptrend in the incidence of BC is being seen among young AIs or ANs, Asians, and NHPIs.
• Non‐Hispanic women initially had a higher incidence of BC but were surpassed by Hispanic women in 2022.
• Overall mortality and LR in young women with BC in the TriNetX network have experienced an increase over the past decade.

## 1. Introduction

Breast cancer (BC) was once historically considered a disease of older women, as the risk for BC increases with age and is highest in those over 65 years old [1]. BC in women under 40 is rare, affecting only approximately 4%–7% of women [1–3]. However, recent studies have demonstrated a significant increase in rates of BC within this younger population [4–6]. BC is now the leading cause of cancer death among women under 40 [7]. These patients often face a poorer prognosis compared to older women due to more aggressive biology and histology, including larger tumors, negative hormone receptor status, and overexpression of human epidermal growth factor receptor (HER2) [2, 5, 7]. Furthermore, screening programs for low‐ to normal‐risk women do not begin until age 40. Thus, most young women who present with BC have locally advanced or metastatic disease at the time of diagnosis. This alarming trend is believed to be partially attributed to lifestyle changes, including increased obesity, fewer live births, and older age at first live birth [7, 8].

Our study aimed to assess the incidence and trend of BC in young women aged 18–40 years old over the past decade and to stratify the results by age, race, and ethnicity. We then evaluated the incidence and trend of mortality and local recurrence (LR) in women aged 18–40 with primary BC. Given the limited data on young women with BC, we hoped to delineate any social or demographic variables contributing to disparities in BC incidence and identify specific populations at risk.

## 2. Materials and Methods

### 2.1. Data Source

The data used in this study were collected on July 20, 2024, from the TriNetX research network’s healthcare organizations (HCOs) U.S. Collaborative Network, a global federated health research network. The TriNetX platform provides access to electronic medical records from over 115 million patients across 66 HCOs. Available information includes demographics, diagnoses (coded using ICD‐10‐CM codes for the International Classification of Diseases, 10th Revision, Clinical Modification), procedures (coded using ICD‐10‐PCS or CPT), medications, and laboratory values. The HCOs comprised hospitals, primary care clinics, and specialists and included patients with or without insurance.

### 2.2. Patient Consent Statement

This retrospective study is exempt from informed consent. The data reviewed are a secondary analysis of existing data, do not involve intervention or interaction with human subjects, and are de‐identified as per the de‐identification standard defined in Section §164.514(a) of the Health Insurance Portability and Accountability Act Privacy Rule. The analysis was conducted solely using aggregated de‐identified patient counts and summary statistics available through the TriNetX platform. Data are directly retrieved from electronic health record systems of participating organizations in a systemic and standardized format.

### 2.3. Design of Cohort

We first created a cohort containing female patients aged 18–40. The clinical event of interest was a diagnosis of BC from January 1, 2014, to December 31, 2023. The ICD‐10‐CM codes used were C50.0 through C50.919.

We then created a second cohort of female patients aged 18–40 years old who were diagnosed with primary BC (ICD‐10‐CM C50.0 through C50.919). The clinical events of interest were mortality (coded as “Deceased” under demographics) and LR (ICD‐10‐CM C77.3 and C79.81).

### 2.4. Statistical Analysis

The Incidence and Prevalence Analytics function on TriNetX was used to assess the incidence proportion of BC in female patients, 18–40 years old, from January 1, 2014, to December 31, 2023. Incidence proportions were stratified by age, race (American Indian or Alaskan Native [AI/AN], Asian, Black or African American, Native Hawaiian or other Pacific Islander [NHPI], and White), and ethnicity (Hispanic or Latino and non‐Hispanic or Latino).

The denominator for each annual incidence proportion consisted of all female patients within this age range available within the TriNetX network during each corresponding calendar year. Because TriNetX is a dynamic, federated database with expanding HCO participation over time, this population at risk is not static, and annual incidence estimates may be influenced by changes in network composition, database growth, and fluctuations in contributing institutions.

Incidence proportion (or cumulative incidence) represents the number of at‐risk individuals who develop a condition of interest within a specified time period, relative to the entire population at risk during that same period. In contrast, the incidence rate measures the occurrence of the condition in at‐risk individuals relative to the total amount of at‐risk person‐time [9]. Given the predefined study period and the inability to obtain reliable person‐time data for individual patients within the federated TriNetX environment, incidence proportion was selected as the most appropriate measure for this analysis.

The TriNetX Incidence and Prevalence tool provides descriptive epidemiologic data only and does not generate confidence intervals, *p* values, or support inferential modeling. As such, sensitivity analyses or hypothesis testing were not performed, and all findings are presented descriptively and should be interpreted as hypothesis‐generating.

## 3. Results

**FIGURE 1:**
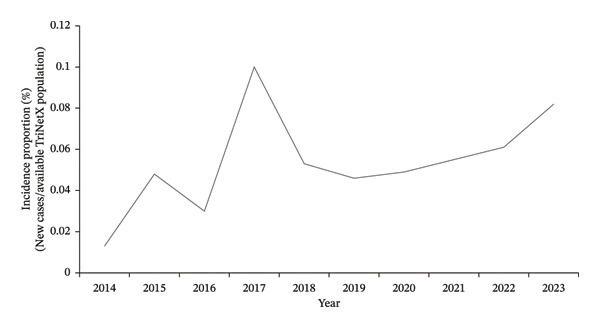
Annual incidence proportion of primary breast cancer in females aged 18–40 (TriNetX network).

18,250,987 female patients between 18 and 40 years old from 66 HCOs were identified in the TriNetX network during the study period. From 2014 to 2023, the incidence proportion of BC in the TriNetX database increased from 0.013% (635 cases) in 2014 to 0.082% (4475 cases) in 2023 (Figure 1). There was a notable peak in 2017, with an incidence proportion of 0.1% and 6621 cases (Figure 1).

When stratified by age, all ages showed an increasing trend of BC. Individuals 30–39 years old had the greatest number of BC cases.

**FIGURE 2:**
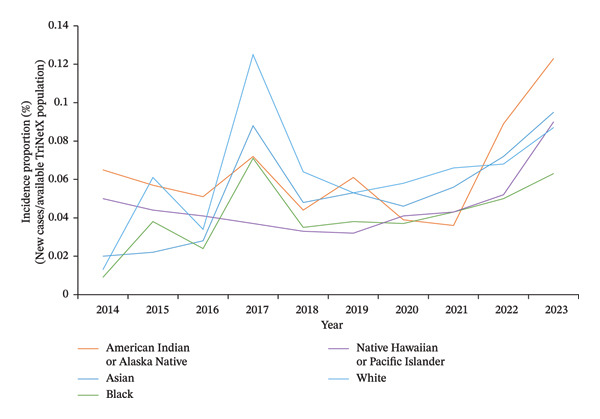
Annual incidence proportion of primary breast cancer in females aged 18–40 (TriNetX network), by race.

Increasing trends were seen across all racial and ethnic groups. When stratified by race, White women had the most cases of BC in each year of analysis; however, incidence proportions of BC showed an uptrend in AI/AN, Asians, and NHPI over the past 4 years (Figure 2). From 2014 to 2023, the incidence proportion increased from 0.009% to 0.063% in Black women, 0.013% to 0.087% in White women, 0.020% to 0.095% in Asian women, and from 0.065% to 0.123% in AI/AN women and 0.050% to 0.090% in NHPI women (Figure 2).

**FIGURE 3:**
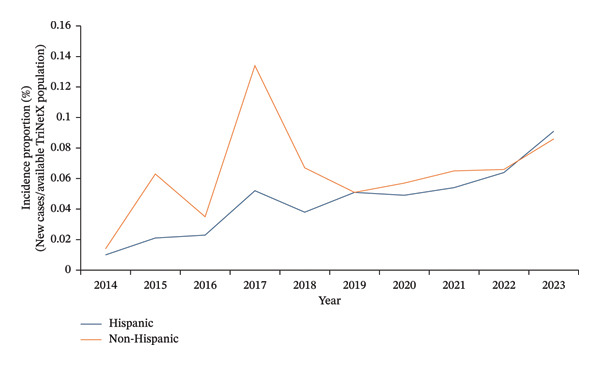
Annual incidence proportion of primary breast cancer in females aged 18–40 (TriNetx network), by ethnicity.

When stratified by ethnicity, non‐Hispanic women initially demonstrated a higher recorded incidence proportion of BC but were surpassed by Hispanic women in 2022. Between 2014 and 2023, the incidence proportion increased from 0.010% to 0.091% in Hispanic women and from 0.014% to 0.086% in non‐Hispanic women (Figure 3).

**FIGURE 4:**
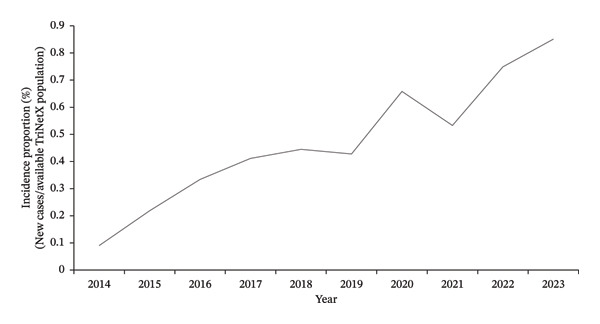
Annual incidence proportion of mortality in females aged 18–40 with breast cancer (TriNetX network).

**FIGURE 5:**
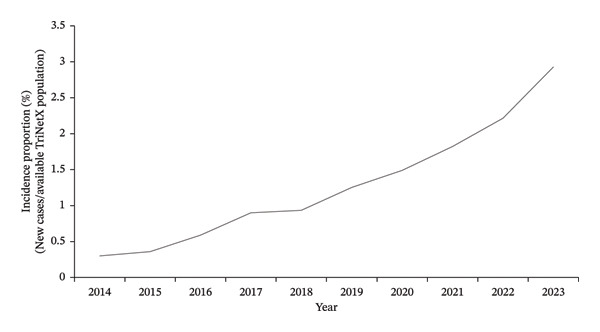
Annual incidence proportion of local recurrence in females aged 18–40 with breast cancer (TriNetX network).

For our second study, we identified 38,683 female patients between age 18 and 40 with a primary BC diagnosis from 63 HCOs. From 2014 to 2023, mortality increased from 0.09% to 0.851% (Figure 4), and LR increased from 0.3% to 2.9% (Figure 5).

## 4. Discussion

Our study identified an upward trend in the recorded incidence proportion of BC among young women over the past decade, which was observed across all racial and ethnic groups within the TriNetX network. The highest incidence proportion of BC occurred in non‐Hispanic White women who were 30–39 years old. While Black and White populations demonstrated the largest absolute increases over the study period, upward trends were also seen among AI/AN, Asians, and NHPI. This aligns with existing research demonstrating that with greater length of U.S. residence, the subsequent generations of immigrants experience increased risk for chronic diseases, especially cancers such as breast, prostate, colorectal, and ovarian cancer [10–12]. This has been demonstrated in studies examining AIs [13], ANs [14], and NHPI [15].

Although non‐Hispanic White women had the highest incidence proportion of BC in our study, prior studies often identified Black women, rather than White, as most affected [4, 6, 16]. When compared to non‐Hispanic women, Hispanic women tend to have higher maternal parity, earlier childbirth, and longer breastfeeding periods [17]. These findings may contribute to their lower incidence of BC, as seen in our study. However, this pattern appears to be changing as fertility rates continue to fall and women are having children at later ages [18, 19]. This is also consistent with our data, which demonstrated a shift in 2022. Furthermore, a migrant study from 2005 found that as Hispanic women spent more time in the United States, their risk of BC increased [20].

The observed peak in 2017 should be interpreted in the context of both biological and database‐related factors. BC incidence proportion peaked in 2017 (Figure 1) across all racial and ethnic groups (Figures 2 and 3), coinciding with the discovery of 72 previously unknown genetic variants that year, including PALB2, TP53, and PTEN [21, 22]. TriNetX also saw a significant data influx during this period as it expanded to multiple HCOs [23].

Despite the overall decrease in female BC mortality rate in the general population due to improved screening and treatment modalities [24], our data demonstrated increasing proportions of recorded mortality and LR among young women over the study period. These findings must be interpreted cautiously, as our analysis was unable to adjust for tumor stage, grade, molecular subtype, or treatment patterns. However, prior literature has demonstrated that young women often present with more advanced disease and biologically aggressive tumors, including larger tumors, higher grade, and higher prevalence of hormone receptor–negative and HER2‐positive subtypes [3, 25].

Young women with BC face unique challenges, including poorer prognosis, higher recurrence risks, and psychosocial challenges, including body image and self‐esteem concerns. For early‐stage BC, evidence suggests that survival is no different between mastectomy and breast‐conserving surgery (BCS) in young women [26–29]. Adjuvant radiation is essential in BCS as these patients have a higher risk of LR when compared to older patients [30, 31]. International consensus guidelines also emphasize that young age alone is not a reason to prescribe more intensive and combination chemotherapy regimens [32]. Endocrine therapy options include tamoxifen, aromatase inhibitors, and the addition of ovarian function suppression (OFS) [33].

It is also important to recognize that amenorrhea and the risk of both temporary and permanent infertility are distinct challenges faced by young women receiving systemic therapy [34]. They are at risk of long‐term health complications, including cardiovascular issues and premature menopause. Many women are often advised to discuss fertility preservation options prior to starting systemic therapy [35]. Optimizing care for young BC patients requires a multidisciplinary approach, including geneticists, fertility specialists, and mental health support.

We acknowledge several limitations of this study due to its retrospective nature and reliance on electronic health records and administrative data across multiple institutions. Residual confounding factors could not be completely avoided due to the nature of the TriNetX platform. For example, we are unable to specify the cause of mortality. Clinical variables such as tumor stage, tumor size, histologic grade, molecular subtype, and receptor status were unavailable, precluding adjustment for biological and treatment‐related factors known to influence prognosis and recurrence. TriNetX is also a growing, dynamic database. Expansion in participating HCOs and improvements in data capture over time likely influenced the number of patients available within each study year. We observed a notable increase in data volume around 2017, consistent with previously documented expansion of the TriNetX network. Consequently, the reported increases in incidence, mortality, and recurrence may partially reflect changes in database composition, improved diagnostic coding, and enhanced longitudinal follow‐up rather than true population–level epidemiologic shifts alone. Finally, formal sensitivity analyses restricted to stable contributing institutions were not feasible within the federated TriNetX environment. Therefore, the temporal trends observed should be interpreted cautiously and considered hypothesis‐generating.

Despite these limitations, the strength of our study lies in the analysis of a large, multicenter, geographically diverse dataset encompassing over 38,000 women under 40 years old with BC. Although formal sensitivity analysis was not feasible, the consistency of observed trends across multiple demographic subgroups suggests that these findings are not solely driven by a single changing population. Our study provides valuable real‐world insight and contributes to the growing body of literature suggesting that, despite improved treatment strategies and screening techniques, BC in young women remains an important and potentially evolving public health concern. Few studies have considered race and ethnicity as integral components of comprehensive BC risk assessment. Large population‐based studies are therefore essential to create stratified analyses of BC risk factors across different regions, especially among AI/AN, Asian, and NHPI populations, to better understand the rising BC incidence in these groups. Potential risk factors to evaluate include oral contraceptive use, alcohol and tobacco use, obesity, diabetes, menopausal status, and breastfeeding. Future research should also include a deeper exploration into the sociodemographic, environmental, and genetic factors that may contribute to disparities in BC incidence, mortality, and screening across racial and ethnic groups.

## 5. Conclusion

The incidence proportion of BC among young women aged 18–40 years old showed an upward trend within the TriNetX network between 2014 and 2023, with the highest number of cases observed among women aged 30–39 years. Increasing incidence proportions were observed across all racial and ethnic groups, with notable recent increases among AI/AN, Asian, NHPI, and Hispanic women. Within this population of young women with BC, increases in recorded mortality and LR were also observed over time.

These findings suggest that adverse outcomes in young women with BC remain an important concern and warrant continued investigation. Larger population‐based and prospective studies with granular information on tumor biology, staging, and treatment are needed to better define true epidemiologic trends, identify high‐risk populations, and guide the development of age‐appropriate risk stratification, screening, and management strategies. Furthermore, ethnicity‐based studies should be considered to better understand disparities in BC incidence and mortality across racial and ethnic groups.

## Author Contributions

Jennifer Den: investigation, writing–original draft, visualization, data curation, and analysis. Nicole Nelson, Raj Vaghjiani, and Douglas Tyler: critical review. V. Suzanne Klimberg: conceptualization, critical review and editing, and supervision.

Jennifer Den had full access to all of the data in this study and takes complete responsibility for the integrity of the data and the accuracy of the data analysis.

The lead author, Jennifer Den, affirms that this manuscript is an honest, accurate, and transparent account of the study being reported; that no important aspects of the study have been omitted; and that any discrepancies from the study as planned (and, if relevant, registered) have been explained.

## Funding

This study received no specific grant from any funding agency in the public, commercial, or not‐for‐profit sectors.

## Disclosure

All authors have read and approved the final version of the manuscript. The research was conducted independently, and no external organization had any role in study design, data collection, analysis, interpretation, or manuscript preparation.

## Conflicts of Interest

The authors declare no conflicts of interest.

## Data Availability

The data analyzed in this study were accessed through the TriNetX network, a federated health research platform that provides de‐identified, aggregated patient data from participating healthcare organizations. Access to TriNetX is institution‐dependent; in this study, data were accessed through the University of Texas Medical Branch (UTMB). No patient‐level data were downloaded, exported, or stored by the authors. Researchers seeking to access similar datasets must request access directly from TriNetX (https://trinetx.com) and comply with both TriNetX data use agreements and their own institutional policies.
